# ZIF-8 Pellets as a Robust Material for Hydrogen Cryo-Adsorption Tanks

**DOI:** 10.1021/acsaem.2c03719

**Published:** 2023-02-01

**Authors:** Rafael Balderas-Xicohtencatl, Jose A. Villajos, Jose Casabán, Dennis Wong, Michael Maiwald, Michael Hirscher

**Affiliations:** † Max Planck Institute for Intelligent Systems, Heisenbergstr. 3, 70569Stuttgart, Germany; ‡ Division Process Analytical Technology, Bundesanstalt für Materialforschung und -prüfung (BAM), Richard-Willstaetter Str. 11, 12489Berlin, Germany; § MOF Technologies Ltd, 63 University Road, BelfastBT7 1NF, United Kingdom; ∥ Advanced Institute for Materials Research (WPI-AIMR), Tohoku University, Katahira 2-1-1, Aoba-ku, Sendai, 980-8577, Japan

**Keywords:** Hydrogen adsorption storage, Reproducibility and standardization, Metal−organic frameworks, ZIF-8, High cycling stability, Volumetric uptake, High usable capacity

## Abstract

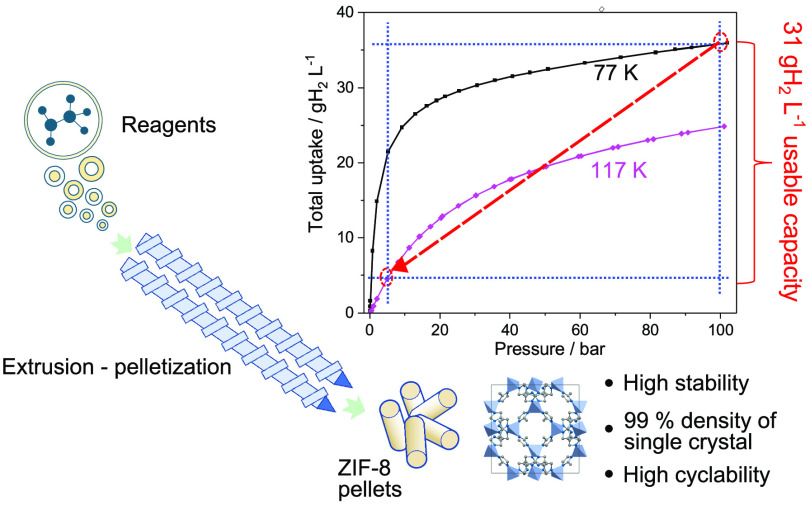

Cryoadsorption on the inner surface of porous materials is a promising solution for safe, fast, and reversible hydrogen storage. Within the class of highly porous metal–organic frameworks, zeolitic imidazolate frameworks (ZIFs) show high thermal, chemical, and mechanical stability. In this study, we selected ZIF-8 synthesized mechanochemically by twin-screw extrusion as powder and pellets. The hydrogen storage capacity at 77 K and up to 100 bar has been analyzed in two laboratories applying three different measurement setups showing a high reproducibility. Pelletizing ZIF-8 increases the packing density close to the corresponding value for a single crystal without loss of porosity, resulting in an improved volumetric hydrogen storage capacity close to the upper limit for a single crystal. The high volumetric uptake combined with a low and constant heat of adsorption provides ca. 31 g of usable hydrogen per liter of pellet assuming a temperature–pressure swing adsorption process between 77 K – 100 bar and 117 K – 5 bar. Cycling experiments do not indicate any degradation in storage capacity. The excellent stability during preparation, handling, and operation of ZIF-8 pellets demonstrates its potential as a robust adsorbent material for technical application in pilot- and full-scale adsorption vessel prototypes.

## Introduction

Hydrogen (H_2_) as an energy carrier is currently stored in compressed (350–700 bar) cylinders and cryogenic (20–30 K) tanks.^1,2^ Depending on the application, mobile or stationary, current storage technologies require higher storage efficiency and safety.^3^ Among solid-state storage solutions, physisorbed H_2_ at cryogenic temperature (i.e., 77 K) on the inner surface of porous materials shows faster kinetics for charging and discharging and complete reversibility compared to chemical hydrides. Besides, cryoadsorption involves lower pressures (typically below 100 bar) compared to compressed gas improving safety and lowering compression costs.^4−6^ Moreover, recent studies explored storage at subcritical temperatures (*T*_c_ = 33 K) and showed reduced boil-off losses using adsorbents compared to liquid H_2_.^7^

Different classes of porous materials have been investigated for H_2_ cryoadsorption for the last four decades, e.g., zeolites and carbons.^8−10^ So far, covalent organic frameworks (COFs) and metal–organic frameworks (MOFs) exhibit the highest porosity and largest BET areas yielding the highest H_2_ storage capacities on a gravimetric basis.^2,11^ Among the different methods to synthesize MOFs,^12,13^ mechanochemical synthesis is feasible for certain structures that use catalytic or even null amounts of solvents like water or ethanol, with clear economic and environmental benefits.^14,15^ It is even possible to extrapolate mechanochemical synthesis into twin-screw extrusion to intensify the production into a continuous process.^16^

For most of the applications, the volumetric storage capacity (amount of H_2_ per unit of volume) is more relevant than the gravimetric uptake and it is calculated using the gravimetric uptake and the adsorbent’s density.^17−19^ Typically, the reported value of the adsorbent’s density is not easy to determine. For crystalline materials such as MOFs, the crystal structure can be determined by XRD, yielding directly the single-crystal density. Generally, polycrystalline powders are obtained instead of perfect single crystals, with a much lower packing density.^20^ Different densification methods, such as extrusion, granulation, or direct compression, can increase the packing density by shaping the powders into granules, monoliths, tablets, or pellets, with or without the addition of binders.^21,22^ On the other hand, too strong mechanical compression of MOF powders may damage their porous structure reducing the surface area and pore volume.^23,24^ As an upper limit of packing density without loss of porosity, values close to the single-crystal density can be realized.^18,20,25^

Material stability and easiness of handling are important parameters considering their lifetime and constant performance as H_2_ stores. Some examples of mechanically and hydrothermally stable MOFs are known, for which the stability depends on structural properties like framework geometry, porosity, and composition, affecting the strength of the metal–organic coordination bound and the linker length and flexibility.^26,27^

A reliable and reproducible measurement of the adsorbent’s H_2_ uptake is fundamental for its large-scale application in, e.g., demonstrators of adsorption tanks with a higher technology-readiness level (TRL).^28^ For example, the gravimetric and volumetric H_2_ uptake of an adsorbent determines the quantity of adsorbent material needed (volume and weight), and the operating temperature and pressure to store a given amount of H_2_. For crystalline materials, an additional quality assessment can be achieved by combining XRD analyses with textural characterization.

ZIF-8 (Zeolitic Imidazolate Framework-8) is a zeolitic-like MOF based on Zn, a cheap metal widely used in industry, and 2-methylimidazole (meIm),^29^ a low-cost linker used in MOF synthesis.^30^ The strong Zn–N coordination bonds within the zeolitic structure provide high thermal and mechanical stability and reduce degradation during handling and activation.^31^ Compared to well-known zeolites, ZIFs show a higher BET area (up to ∼1800 m^2^ g^–1^)^32^ because of the larger pore size and, therefore, a higher H_2_ uptake. Furthermore, the high hydrophobicity of ZIF-8 reduces the adsorption of moisture during handling in air, e.g., filling of adsorbent material in the storage tank.^31^

In this work, pellets and powders of ZIF-8 were synthesized mechanochemically by twin-screw extrusion. High-precision H_2_ adsorption measurements were performed on powder and pelletized samples up to 100 bar in two different laboratories using three different experimental setups. For this, we developed calibration procedures and analysis protocols specific to each analyzer, considering the common experimental deviations during adsorption measurements and compensating for the effect of thermal gradients at low and high pressures. The structural stability and the H_2_ adsorption–desorption operation after pelletizing were compared to the powder. The volumetric uptake of both powder and pellets was calculated using the packing density after tapping and the average density of single pellets.

## Materials, Methods, and Calculations

### Synthesis and Characterization

Powders and pellets of ZIF-8 were synthesized and pelletized by MOF-Technologies using a continuous mechanochemical process carried out by reactive extrusion^16^ at 150 rpm screw speed using a Kraus Maffei Berstorff 25 mm TSE that enabled a production rate of 1.3 kg/h. For the synthesis, an additive is added together with the reagents, whose nature is protected by IP. After being manufactured, the products were outgassed to remove solvent traces and stored in closed bags under vacuum. Powder X-ray Diffraction patterns (PXRD), as structural characterization, were collected using Cu K_α_ radiation (λ = 1.50406 nm) on a D8 Advanced diffractometer (Bruker AXS, Germany) equipped with a Lynxeye detector. Samples were measured in reflection geometry in a 2θ range from 3° to 50° with a step size of 0.009°. The textural characterization was performed by N_2_ adsorption–desorption experiments at 77 K using a volumetric device (ASAP 2020 Micromeritics). The experiments were performed using 150 mg of sample in the range of relative pressures *p*/*p*_0_ from 10^–7^ to 0.996 and controlling the temperature by submerging the sample in liquid N_2_. BET analysis for calculating the surface area was performed using a relative pressure range (0.001–0.005) of the N_2_ isotherm. The BET range was chosen following Rouquerol and Llewellyns’ recommendations^33^ and considering the structural transition of ZIF-8. The total pore volume (*V*_p_) was calculated from the N_2_ uptake at *p*/*p*_0_ ≈ 0.90.

### Hydrogen Adsorption Measurements

An automated Sieverts-type apparatus (Setaram-HyEnergy PCTPro-2000, see Figure S2 in the ESI) was used to measure the hydrogen excess uptake at cryogenic temperature and up to 25 bar. A microdoser (MD) from HyEnergy is capable of accurately measuring the storage properties using small amounts of sample (∼100 mg). HPVA-II (High-Pressure Volumetric Analyzer) from Micromeritics was used to perform experiments up to 100 bar. The larger volume of the manifold and analysis cell requires a larger amount of sample (0.6 to 2.5 g depending on the sample cell) for good accuracy. In both devices, the cryogenic temperature was controlled by the described systems in Figures S2 and S6 in the ESI. The measured adsorption data were corrected by the corresponding calibration procedure described in section 2.4 of the ESI compensating the temperature gradients between the sample cell at low temperature and the apparatus at room temperature. High-pressure adsorption experiments were also collected with a gravimetric device (XEMIS fabricated by Hiden Isochema) that uses a microbalance to measure the change in weight in a sample. The gas pressure was dynamically controlled from 0 to 120 bar and recorded by three different pressure transducers with the ranges (0.001–1) ± 0.0005, (0–20) ± 0.01, and (0–200) ± 0.1 bar. The analysis temperature was controlled by submerging the reactor in a Dewar with liquid N_2_ or Ar. Measured data do not require correction for temperature gradients but do require correction for the effect of sample buoyancy (see section 3.2 in the ESI). Highly pure H_2_ (99.999% in HPVA, 99.9999% in PCT and XEMIS) and He (99.9999%) were used for analysis or volume determination, respectively.

The experimental measurements of the H_2_ uptake described above provide the excess uptake (*n*_exc_) of the adsorbent materials, given by the total amount of gas contained in the pores minus the amount of gas that would be present in the pores in the absence of gas–solid intermolecular forces.^9,17^ The actual amount of adsorbed phase is called absolute uptake (*n*_abs_) and it is calculated from eq 1 under the assumptions: (i) The density of the excess surface amount is equal to the bulk liquid density of the adsorbate (ρ_liq_); (ii) the volume of the excess surface is . The total uptake (*n*_total_) is calculated by adding the number of gas molecules (nonadsorbed) in the pore volume (*V*_p_) to the excess uptake (eq 2) and describes the actual storage capacity of the adsorbent.
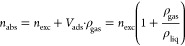
1
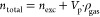
2

Here, we assumed a H_2_ liquid density is ρ_liq_ = 70.8 kg m^–3^ and the bulk gas density ρ_gas_ is given by the equation of state for H_2_ at the corresponding pressure and temperature values.^34^

**Figure 1:**
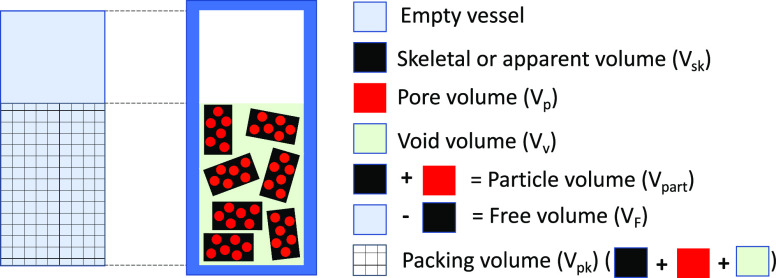
Volume definitions in an adsorption vessel.

Figure 1 shows the different volume definitions in an adsorption system.^17^ The apparent or skeleton volume of materials (*V*_sk_) refers to the solid framework of the adsorbent. The particle volume (*V*_part_) is equal to the pore volume (*V*_p_) plus *V*_sk_ and corresponds to the volume of the outer size of the adsorbent, in our case, a MOF powder particle or a pellet. The packing volume (*V*_pk_) is obtained when the void volume (*V*_v_) between the particles is added to the particle volume (*V*_part_).

The total volumetric uptake was calculated from the excess and the corresponding density values using eq 3.^35^ Note that with this equation, different volumetric uptakes can be calculated depending on the considered volume or density (ρ), i.e., the density of the particles, the crystallographic density, or the packing density (see Figure 1).
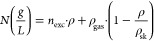
3where *N*(g/L) refers to the volumetric storage density of both the adsorbed phase and the compressed gas phase.

The packing density of the powder or pellets was determined using the tapping method. A known mass of material is filled in a cylinder (in our case 20 g and a diameter of 50 mm) and after tapping the filled cylinder 30 times, the height of the filling is measured. Also, the density of the pellets is calculated by measuring the mass, radius, and length of 45 individual pellets, assuming a cylindrical shape, and finally averaging the values (see Table S1 and Figure S1 in ESI).

## Results and Discussion

**Figure 2:**
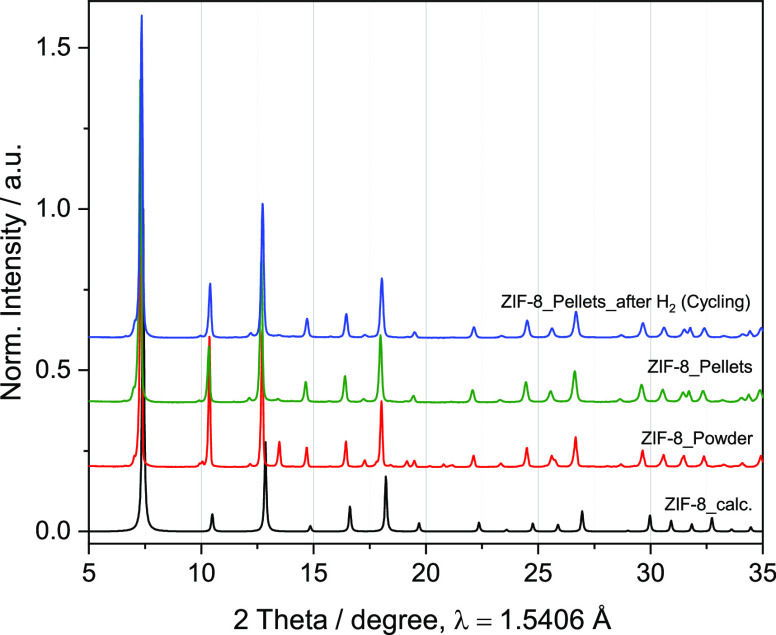
PXRD of the powder and pelletized ZIF-8 samples before and after the adsorption/desorption experiments of H_2_.

ZIF-8 was mechanochemically synthesized by MOF Technologies using a reactive extrusion process allowing the scale of the synthesis of the material to large quantities. PXRD confirms that the powders and pellets are composed completely of ZIF-8 crystalline phase (Figure 2) that corresponds to the phase reported by Morris et al. (C_24_ H_30_ N_12_ Zn_3_) with a space group *I4̅3m* and unit cell dimensions *a* = *b* = *c* = 17.0095(8) Å.^36^ Using the unit cell dimensions and the chemical formula, the crystal density is calculated to 0.921 g cm^–3^. The diffractograms are composed of sharp peaks at 2θ diffraction angles ca. 7°, 11°, 13°, 15°, 17°, and 18°. In all cases, the peaks correspond to the simulated pattern from the theoretical crystalline phase (ZIF-8_calc.). Additional signals at 13.5°, 17.2°, and 19.1° were also observed for all materials investigated, which can be related to additives used in the synthesis. The pellets maintain the ZIF-8 phase with high crystallinity and a lower relative intensity of corresponding reflections to the additive, potentially ascribed to a higher crystallinity of the sample after the postprocessing step.

**Figure 3:**
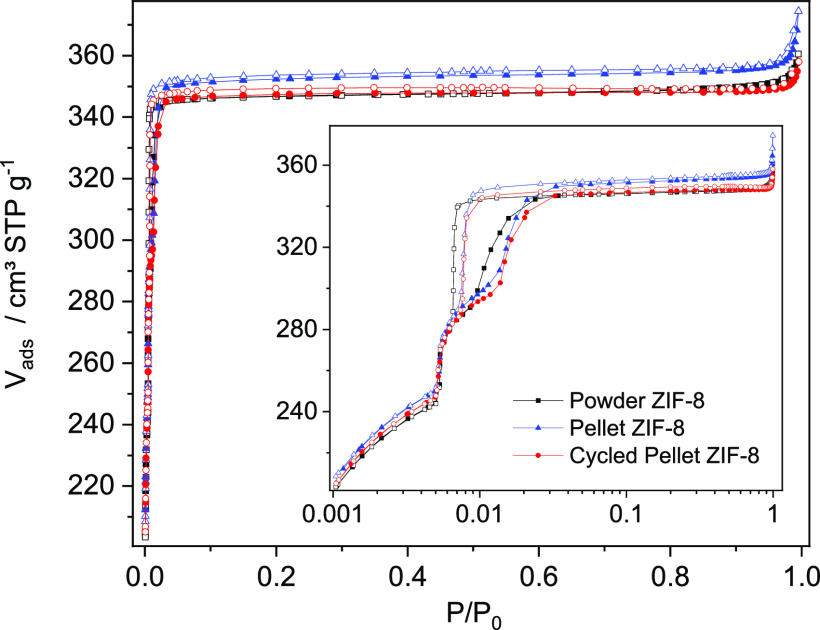
N_2_-adsorption/desorption isotherms at 77 K of powder and pelletized ZIF-8 samples.

N_2_ adsorption–desorption experiments at 77 K on powder and pelletized ZIF-8 (Figure 3) show type I(a) adsorption isotherms according to the IUPAC classification corresponding to ultramicroporous materials.^33^ The isotherms show hysteresis during desorption, which was attributed to framework flexibility due to a gate-opening effect involving the linker molecules.^37^ The pellets show higher uptake at *p*/*p*_0_ values ca. 0.01 compared to powder, indicating a slightly higher BET area (ca. 2%, see Table 1).

**Table 1:** Textural Properties of the Powder and Pelletized ZIF-8

Sample	*A*_BET_/m^2^ g^–1^	*V*_p_^a^/cm^3^ g^–1^	*C*
ZIF-8 Powder	1115	0.54	3634
ZIF-8 Pellets	1142	0.55	3550
Cycled ZIF-8 Pellets	1128	0.54	3553

a Total pore volume at *p*/*p*_0_ = 0.9.

The excess H_2_ uptake at 77 K of powder and pelletized ZIF-8 was measured by two volumetric and one gravimetric apparatus. The volumetric measurement is based on the change of pressure when a reservoir with known volume is connected to a sample chamber. Calculating the adsorbed amount requires accurate knowledge of the free volume (*V*_F_), which is the empty vessel volume minus the skeleton volume of the sample (see section 2.3 in the ESI), necessary to calculate the adsorbed amount. On the other hand, a gravimetric apparatus measures the sample weight providing a direct and continuous measurement of the excess adsorbed amount. In this case, the measured weight requires correcting the buoyancy of the sample, which is directly related to the skeleton volume (*V*_sk_, see section 3.2 in the ESI). In both cases, the skeleton volume is determined by an independent He experiment conducted at room temperature and low pressures for the volumetric apparatus and the full-pressure analysis range for the gravimetric one. The accuracy of the measurement of this volume is fundamental for the accuracy of the H_2_ uptake measurement, especially at high pressure, where an error of 1% in the determination of this free space can propagate an error of up to 25% in the adsorption calculations at 80 bar.^38^

**Figure 4:**
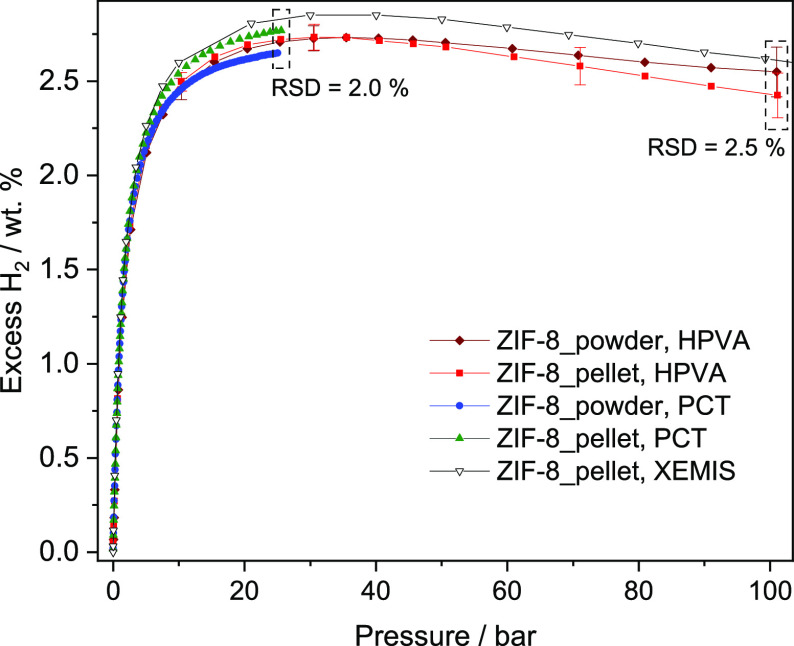
Excess H_2_ uptake at 77 K of ZIF-8 in powder or as pellets.

Figure 4 shows the adsorption isotherms of H_2_ at 77 K of the analyzed samples. In general, the higher the pressure, the higher the excess uptake, reaching a maximum uptake called saturation at a pressure value of about 35 bar. From this maximum, a further increase in pressure results in a negative increment of the excess uptake because the density of the gas phase increases with pressure but the adsorbed phase, at supercritical temperature, behaves as an incompressible fluid.^39^ The analyzed materials adsorb 2.64–2.82 wt % at 25 bar in line with Chahine’s Rule, which predicts ca. 1 wt % of excess uptake at intermediate pressure per 500 m^2^ g^–1^ of BET area.^9,39^ The relative standard deviation (RSD), which evaluates the overall deviation of the measurements with respect to the average value, is 2% at 25 bar among measurements of powder and pellets. In comparison, the RSD from analyzing seven fresh ZIF-8 powders in the same device is 1% at 25 bar (see Figure S7 in ESI), which is close to the previous value, and also similar to the previous interlaboratory measurement of H_2_ cryoadsorption (1–4% involving 15 laboratories).^40^ These results indicate a good reproducibility of the measurements between the different analyzers, and also, a good homogeneity of the adsorption uptake of the different fractions of analyzed samples.

The RSD at 100 bar comparing powder and pellets is 2.5%, similar to the value at 25 bar (2%), and to that for the powder in one analyzer (2.4%). This similarity indicates that the measurement of the free space and the H_2_ uptake, as well as the calibration procedures for all devices, have been successfully performed, but also other experimental conditions, e.g., temperature gradients were carefully corrected. First, the resolution of the H_2_ isotherms is highly dependent on the amount of analyzed sample. The larger the amount of sample, the higher the adsorbing surface that is responsible for a more significant change in the pressure reduction (volumetric measurement) or the weight change (gravimetric measurement) due to the adsorption of more adsorbate molecules. In this work, the amount of sample was used to ensure a figure of merit higher than 1,000% for small pressure steps below saturation, according to recommendations.^28,41,42^ The amount of sample can be also changed to identify systematic errors during analysis due to wrong calibration of the temperature or pressure sensors. Here, different sample amounts of ZIF-8 were used for the three devices: 150–250 mg in the PCT, 0.6–0.8 g for powder and 1–3 g for pellets in the HPVA, and 30–60 mg in XEMIS. In all cases, the measured uptakes are comparable (see Figure 4 and the H_2_adsorption isotherms in ESI) and no systematic error can be observed.

The gas purity of the H_2_ and He greatly impacts the quality of the adsorption results depending on the types of gas contaminants, sample, and technique. Regardless of the hydrophobicity of the sample, almost any gaseous contaminants will preferentially adsorb compared to H_2_ due to their stronger interaction with surfaces.^43^

The activation of the sample before the experiments is usually conducted under dynamic vacuum at a given temperature. Proper activation removes previously adsorbed species from the sample surface and assures a clean inner surface available for H_2_ adsorption. In this work, we studied the effect of the activation time by comparing the adsorption uptake of the material after 4 and 18 h of activation, however, no significant difference could be observed (see Figure S3 in ESI).

At room temperature and moderate pressure, H_2_ and helium approximate an ideal gas, with a compressibility *Z* ≈ 1. However, at higher pressure or lower temperature (∼77 K), H_2_ can no longer be treated as an ideal gas, which is reflected in a nonlinear behavior of the gas density as a function of the pressure. The correct density of the gases can be obtained from databases or from the appropriated EOS as a function of temperature and pressure.^34^

For the measurements at low temperature and high pressure, where the instrument and dosing volume are typically at room temperature and the sample cell is at cryogenic temperature, the nonlinear dependence between density and temperature creates further complications because of the appearance of thermal gradients between sections. This unknown thermal gradient between the instrument and sample cell includes uncertainty in the calculation of the gas density or the buoyancy correction and, therefore, the calculated *n*_exc_. Generally, the deviation of the measured gas uptake due to these thermal gradients is more significant at high pressure.^44^

The correction of this temperature gradient for measurements at cryogenic temperature is especially relevant for the comparability of the results. Here, we present two independent measurements with two volumetric apparatus that applies different correction procedures to account for the nonadsorbed gas in a volume with a thermal gradient. For the PCTPro-2000, the H_2_ excess uptake (*n*_exc_) of the sample is corrected, for each pressure step, by subtracting the amount of gas in the analysis cell with adsorbing sample (*n*_exc_ + *n*_gas_) minus the equivalent amount of gas within the same free space (*n*_gas_), without adsorbing sample, at the same analysis temperature profile (see details in section 2.4 in ESI).

For the HPVA, *n*_exc_ is corrected by using a corrected value of the free volume at the analysis temperature (see the procedure in section 2.4 in ESI). For this, the free volume at the analysis temperature of a blank experiment (empty vessel) is modified so that the adsorption uptake is balanced around zero adsorption. After that, the corrected free volume at the analysis temperature with a sample is calculated by subtracting its skeleton volume (*V*_sk_) from the blank analysis free volume.

**Figure 5:**
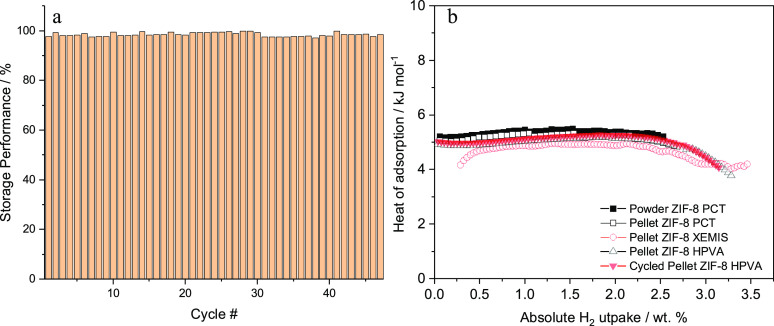
Cycling adsorption–desorption experiments of ZIF-8 pellets. The adsorption performance for each cycle was calculated with respect to the highest measured loading at the maximum storage pressure (a). Isosteric heat of adsorption of ZIF-8 as powder, pellets, and after cycling adsorption–desorption test (b).

The stability of ZIF-8 to H_2_ adsorption–desorption was tested by cycling 47 times from 10 to 86 bar. Due to experimental limitations, the cycling test was performed on different days with an intermediate activation of the adsorbent. In each cycle, the adsorption performance refers to the excess uptake at 86 bar with respect to the highest uptake measured for the material throughout the 47 measurements. The storage performance of the material does not change along with the cycling experiment (Figure 5a) due to the structural stability of the material to the adsorption–desorption operation (see Figure 2). The slight differences between cycles are due to the different maximum pressure values at which the uptake is measured in the different cycles (different pressure values mean differences between the initially dosed and the previously remaining gas in the material).

The heat of adsorption (see section 4 of the ESI for calculation) for all samples (powder, pellets, and after cycling) is close to 5 kJ mol^–1^ and remains almost constant with surface coverage (Figure 5b). This constant heat of adsorption indicates homogeneity among the adsorption sites distributed over the surface, which means a proportional heat flow during almost any amount of loaded or released H_2_. The heat of adsorption only decreases after loading more than 2.2 wt % of absolute uptake. Comparing powder, pellets, and the pellets after the cycling experiment, the variation is ±0.5 kJ mol^–1^, confirming that the interaction energy does not change after pelletizing nor during operation.

Besides the gravimetric uptake, the volumetric storage capacity is relevant for the characterization of an adsorbent material with respect to technical applications.^18^ The volumetric uptake can be calculated using different volume definitions (see Figure 1). The crystal density is 0.92 g cm^–3^, obtained by XRD.^36^ The packing density of the powder and pellets was measured by tapping in a cylinder recipient using a large amount of sample (i.e., 20 g of pellets, equivalent to ∼1200 pellets averaging the pellets sizes collected in Table S1 in ESI). The packing density of the tapped pellets was measured as 0.44 g cm^–3^, which is twice as 0.27 g cm^–3^ of the tapped powder. Despite the evident interpellet volume, almost double of adsorbent is weighed in the same volume of pellets compared to the powder. Furthermore, the average density of the individual pellets was calculated based on the size and weight of 45 pellets (Table S1 and Figure S1 in ESI) to 0.9 ± 0.1 g cm^–3^, which is - within the error - similar to the single crystal density.

**Figure 6:**
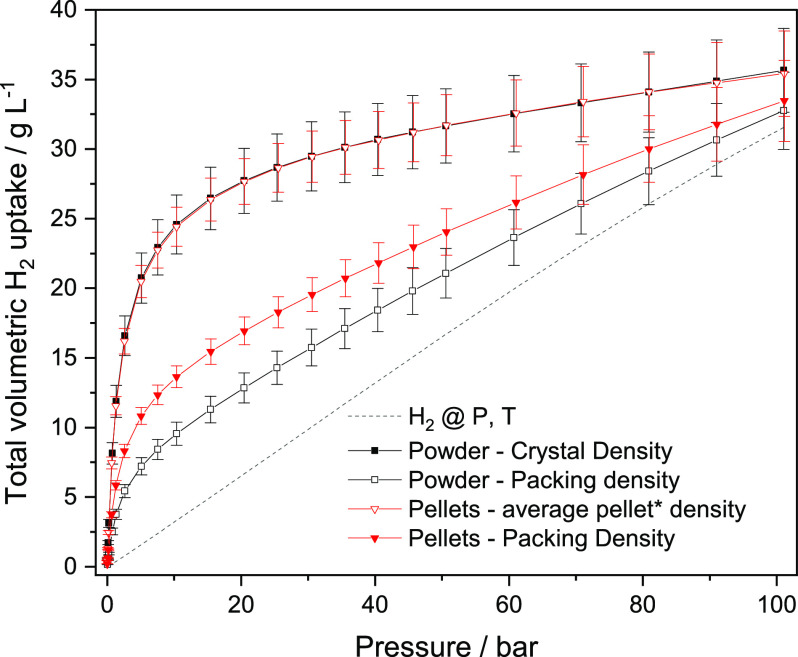
Volumetric uptake of ZIF-8 at 77 K considering different density values.

Figure 6 shows the total volumetric H_2_ isotherms at 77 K for ZIF-8 pellets and powders using the crystal density, the packing density, and the pellet density. Based on the pellet density, a total volumetric capacity near 35 g L^–1^ can be reached at 100 bar and 77 K, which is comparable to the volumetric capacities of MOFs with higher surface area.^45−48^ The extrusion process has the potential to reach a density almost as high as the single-crystal density without loss of surface area, providing a high volumetric capacity involving a potentially cheap, stable, and scalable MOF.

**Figure 7:**
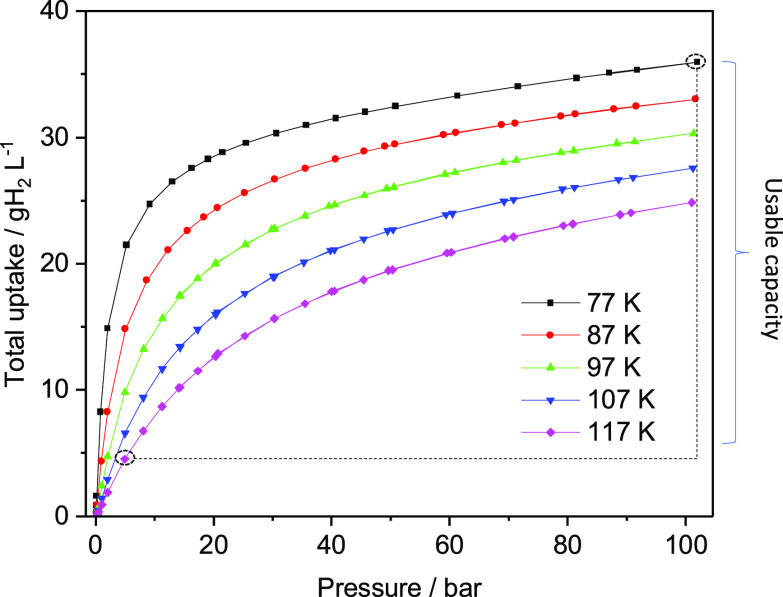
Usable capacity of pelletized material ZIF-8 calculated for a TSA cycle from 77 K – 100 bar and 117 K – 5 bar.

Figure 7 and Figure S13 from the ESI show the usable capacity of H_2_ on a volumetric and gravimetric basis, respectively,^49^ for pellets of ZIF-8 calculated assuming a temperature–pressure swing adsorption process (TSA) between 77 K – 100 bar and 117 K – 5 bar. Using the density of pellets, the volumetric usable capacity is 31.4 gH_2_ L^–1^ (36.9 gH_2_ kg^–1^ is the gravimetric one). Based on this usable capacity under the mentioned temperature and pressure swing, reversible storage of 5 kg of H_2_ will require a volume of 160 L. Extending the temperature swing from 117 to 160 K, the usable capacity of ZIF-8 will be even higher. With this extended temperature swing, other materials like MOF-5, MOF-177, NU-1103, -1101, -1102, and -125; UIO-67 and -68; HKUST-1, PCN-250, and Cu-MOF-74 show a higher usable capacity near 40 gH_2_ L^–1^.^19,50−52^ However, these materials are in general built from more expensive building blocks or show problems of stability during storage or operation. For comparison, the deliverable uptake of a commercial compressed gas cylinder operating at room temperature and within the pressure range 700–5 bar is 38.8 gH_2_ L^–1^, but operating at seven times higher pressure.

## Conclusion and Outlook

ZIF-8 powder and pellets have been synthesized by mechanochemical twin-screw extrusion and both show basically the same crystal structure and porosity, confirming the mechanical stability of the framework and the high degree of control over the synthetic process. The H_2_ adsorption uptake at 77 K was measured with high accuracy considering the amount of analyzed sample, gas purity, activation conditions, and calibration procedures. The relative standard deviation (RSD) of 14 independent H_2_ measurements is 2% at any pressure (up to 100 bar), indicating excellent reproducibility between the used analyzers. The operation stability of the material was demonstrated after 47 adsorption–desorption cycles. Furthermore, the heat of adsorption was measured as ∼5 kJ mol^–1^ remaining almost constant over most of the usable capacity range. This type of interaction assures a proportional heat flow during almost any amount of loaded or released H_2_. Additionally, ZIF-8 pellets show high volumetric total capacity (∼35 g L^–1^) near the theoretical maximum (corresponding to a single crystal) without loss of gravimetric capacity compared to powder. The corresponding volumetric usable capacity of ZIF-8, assuming a TSA operation from 77 K – 100 bar to 117 K – 5 bar, is 31.1 gH_2_ L^–1^, among the highest usable capacities reported so far considering the measured packing densities of MOFs, which would be sufficient for operation in a H_2_ stationary application. Also, the stability of the material is high during preparation, handling, and operation. The heat of adsorption is almost constant throughout the surface coverage of the material. This allows an easier calculation/design of the heat-exchange system necessary during the adsorption/desorption operation. The composition of the material is based on Zn, one of the most abundant metals, and 2-methylimidazol, a comparatively low-cost linker used for the synthesis of MOFs. Typically, the synthesis of MOFs requires harsh conditions and large amounts of hazardous solvents during synthesis. In comparison, ZIF-8 can be produced by mechanochemical approaches that are practically solvent-free methods fulfilling one of the most important principles of green chemistry. Furthermore, a continuous process like the twin-screw extrusion used herein allows the massive production and cost reduction of the material, which is necessary for a large-scale utilization of H_2_ adsorbents. For all these reasons, we remark on the potential of these pellets of ZIF-8 as an adsorbent material for upscaling H_2_ cryo-adsorption technology into prototypes such as in the projects MOST-H2^53^ or AMBHER,^54^ or even on a larger scale.

## Supporting Information Available

Density measurements, hydrogen adsorption results and devices description, fundamentals of the correction and calibration procedures, and calculations of the heat of adsorption (PDF)

## Abbreviations

BET: Brunauer–Emmett–Teller
COF: covalent–organic framework
IUPAC: International Union of Pure and Applied Chemistry
MD: microdoser
MOF: metal–organic framework
PCT: pressure–composition isotherms
PXRD: powder X-ray diffraction
RSD: relative standard deviation
TSA: temperature–pressure swing adsorption
XRD: X-ray diffraction
ZIF: zeolitic imidazolate framework
