# Supplementary material for: ZIF-8 Pellets as a Robust Material for Hydrogen Cryo-Adsorption Tanks
Source: ACS Appl Energy Mater. 2023 Feb 1;6(18):9145–52. doi: 10.1021/acsaem.2c03719 (PMC10523355; doi:10.1021/acsaem.2c03719)
Supplement: Supplementary file 1 — ae2c03719_si_001.pdf [file ae2c03719_si_001.pdf]

Supporting Information:

ZIF-8 pellets as a robust material for hydrogen cryo-adsorption tanks

Rafael Balderas-Xicohtencatl[1,‡], *Jose A. Villajos*[2, ‡], *Jose Casabán*[3], *Dennis Wong* [3], *Michael Maiwald*[2]*, *Michael Hirscher*[1,4]*

[1]Max Planck Institute for Intelligent Systems, Heisenberg Str. 3, Stuttgart, Germany.

[2]Division Process Analytical Technology, Bundesanstalt für Materialforschung und -prüfung (BAM), Richard-Willstätter Str. 11, Berlin, Germany.

[3]MOF Technologies Ltd, Belfast, 63 University Road, U.K.

[4]Advanced Institute for Materials Research (WPI-AIMR), Tohoku University, Katahira 2-1-1, Aoba-ku, Sendai, 980-8577, Japan.

‡*Equal contribution*

**Corresponding: michael.maiwald@bam.de, hirscher@is.mpg.de*

1. Pellet density measurement

The density of the pellets (ρ^{pellet}) was calculated assuming a cylindrical shape and dividing the mass of each pellet by the volume given by the diameter and the length.

$$\rho^{pellet} = \frac{m_{pellet}}{\pi/_4\, d^2 \cdot L}$$

Table S1. Measured dimensions, weight, and calculated density of some pellets.

#	Mass / mg	Diameter / mm	Length / mm	Density / g cm^{-3}	#	Mass / mg	Diameter / mm	Length / mm	Density / g cm-3
1	15.8	1.65	7.70	0.96	24	10.4	1.80	5.20	0.79
2	20.1	1.60	9.85	1.02	25	10.0	1.60	4.75	1.05
3	23.0	1.80	11.60	0.78	26	21.3	1.65	10.10	0.99
4	23.7	1.70	10.70	0.98	27	22.9	1.60	10.65	1.07
5	12.8	1.80	6.35	0.79	28	13.7	1.75	6.95	0.82
6	22.6	1.70	10.00	1.00	29	13.1	1.60	6.30	1.03
7	14.9	1.60	7.20	1.03	30	18.8	1.75	9.00	0.87
8	19.1	1.80	9.50	0.79	31	14.3	1.70	7.10	0.89
9	12.9	1.80	6.75	0.75	32	12.6	1.70	6.25	0.89
10	13.1	1.75	6.40	0.85	33	11.2	1.70	5.50	0.90
11	14.6	1.70	7.00	0.92	34	10.7	1.70	5.45	0.87
12	15.9	1.70	7.70	0.91	35	17.2	1.70	8.25	0.92
13	21.2	1.65	10.10	0.98	36	13.2	1.60	6.35	1.03
14	10.6	1.65	5.15	0.96	37	13.3	1.70	6.50	0.90
15	19.8	1.70	9.80	0.89	38	18.2	1.70	8.75	0.92
16	20.1	1.70	9.50	0.93	39	16.3	1.70	8.00	0.90
17	19.1	1.70	8.80	0.96	40	14.8	1.70	7.00	0.93
18	10.4	1.65	5.20	0.94	41	17.4	1.70	8.55	0.90
19	17.8	1.75	8.90	0.83	42	15.8	1.80	8.05	0.77
20	16.5	1.65	7.55	1.02	43	23.8	1.65	10.65	1.05
21	22.0	1.65	10.05	1.02	44	23.7	1.75	11.50	0.86
22	15.0	1.60	6.90	1.08	45	18.4	1.65	8.55	1.01
23	14.6	1.70	6.75	0.95					

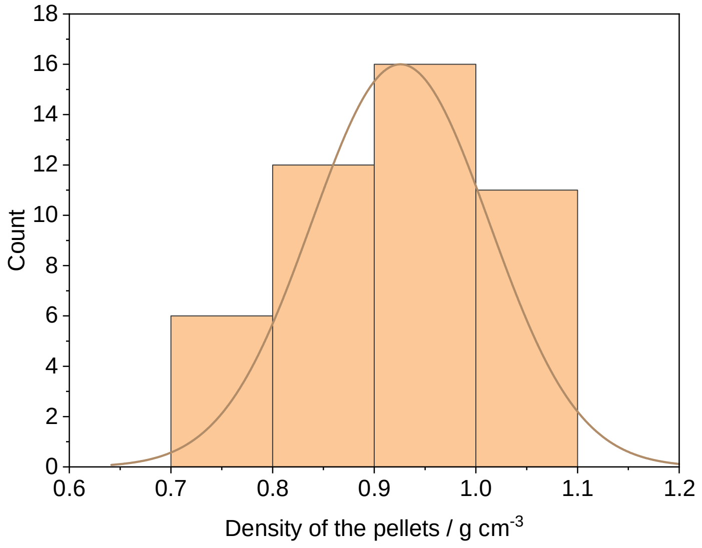

Figure S1. Histogram for individual pellets''' measured density.

2. Measurement of hydrogen uptake by volumetric devices

2.1. PCT-Pro

Figure S2 shows the schematic illustration of PCTPro2000 setup (Setaram-HyEnergy PCTPro-2000). The original setup was coupled with an in-house made cooling-heating device regulated by a temperature controller. This configuration allows us to perform hydrogen adsorption measurements in a wide temperature range (77 to 300 K). For isotherms at 77 K and 87 K, the sample holder is submerged in liquid nitrogen or argon, respectively (Figure S2a). For temperatures above 87 K, the sample container is enclosed by a copper block (orange) with a cooling finger submerged in liquid nitrogen (Figure S2b). The Activation of the samples was performed at 120 °C during 4 – 18 h under dynamic vacuum ($P < 10^{-6}$ mbar) with no difference in the adsorption uptake (Figure S3).

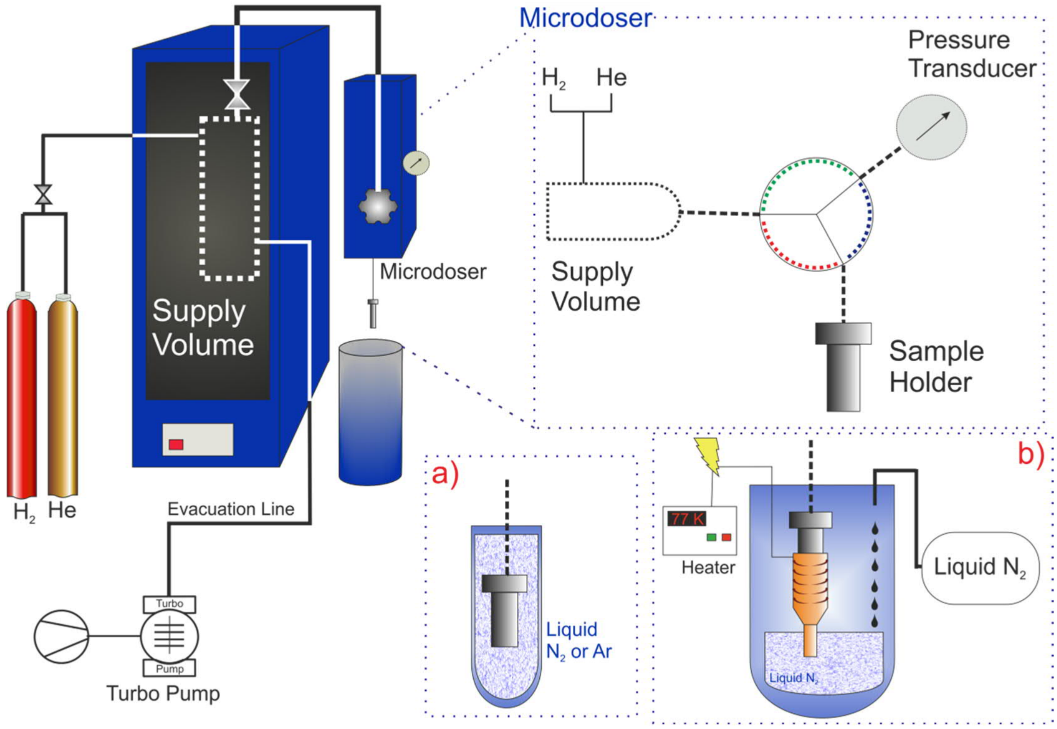

Figure S2. Schematic diagram of the Sieverts´ apparatus (PCT-Pro) coupled with a cooling system (77 K-300 K) a) Dipping method with Dewar cryostat for cooling at liquid nitrogen (77 K) and liquid argon (87 K) temperature. b) Cooling system for temperatures above 87 K where the sample container is enclosed by a copper block (orange) and partially sank in the liquid nitrogen. The liquid nitrogen level is constantly controlled by an automatic liquid N_2 refilling system.

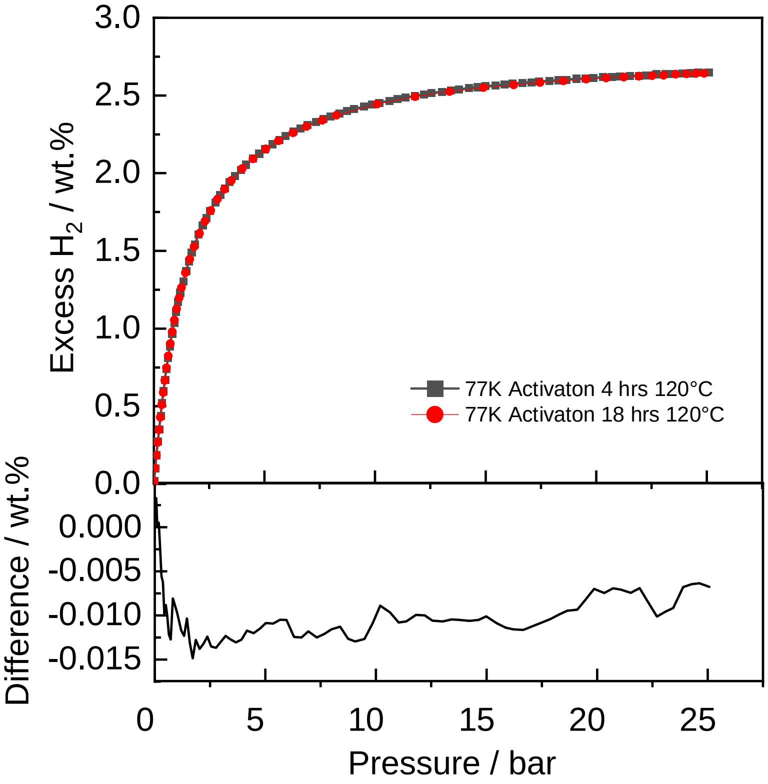

Figure S3. Effect of the activation time in the H_2 adsorption uptake at 77 K.

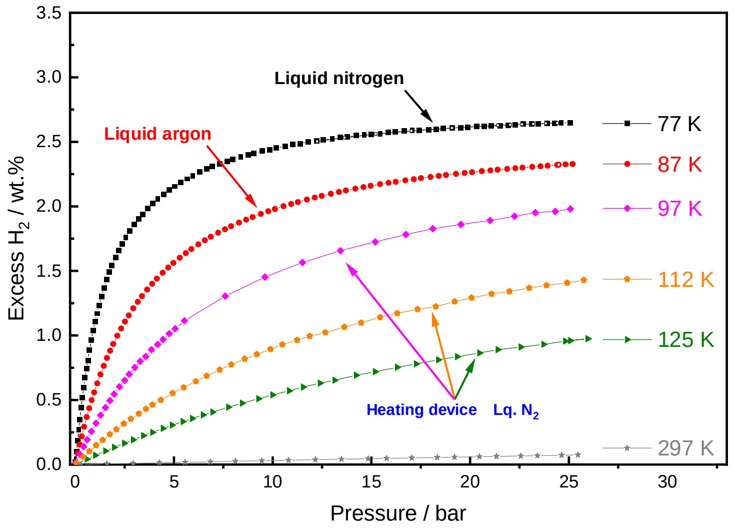

Figure S4. Excess adsorption isotherms at different temperatures of the powder of ZIF-8.

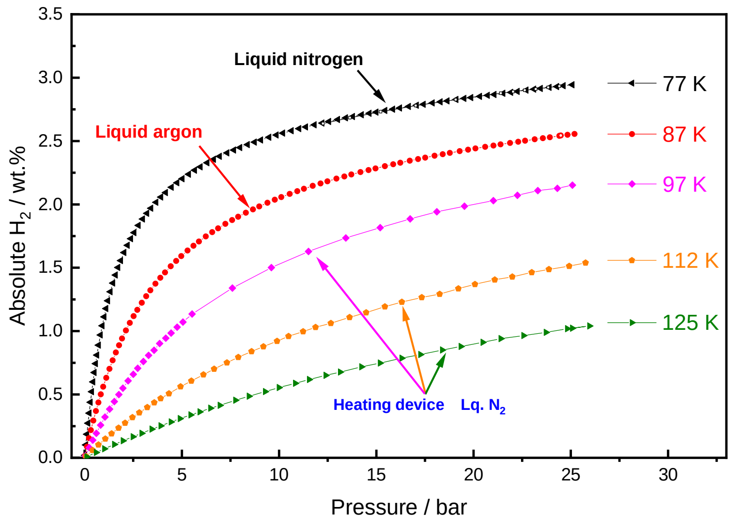

Figure S5. Absolute adsorption isotherms at different temperatures of the powder of ZIF-8.

2.2. HPVA II

The HPVA II (high-pressure volumetric analyzer) uses two pressure transducers for reading the manifold's pressure up to 1 bar (accuracy 0.15 % of the reading) and up to 200 bar (accuracy 0.04 % of the full-scale). For a typical experiment, enough amount of material was used to assure the figure of merit is higher than 10 at low pressures below the adsorbent´s saturation.[1] Adsorption equilibration was assured by using 20 min of equilibration time in all the adsorption steps. The reported excess uptake was averaged from different measurements with an intermediate evacuation for at least six hours at room temperature. For analysis, H_2 gas at 99.999 % and He at 99.9999 % were used. Densities for He and hydrogen gases were calculated from P and T values by MBWR EOS of McCarty and Arp and Helmholtz EOS, respectively, as recommended by NIST.[2, 3] The controlled room temperature is stable within ± 1 °C, and the manifold temperature is kept at 32–33 °C and continuously monitored. The analysis temperature is controlled by using a bath with liquid nitrogen for experiments at 77 K (Figure S6a). A cryo-jacket, provided by Micromeritics, keeps the level of liquid nitrogen in contact with the sample tube approximately constant despite the evaporation of this liquid. When different cryogenic temperatures are needed, the cooling head shown in Figure S6b is used as a cryostat. In this device, previously compress helium exchanges heat with the cold outcoming helium at low pressure from the cooling head. This cold and

compressed gas iso-entropically expands to further cooling the cooling head and is therefore exhausted back to the compressor, previous heat exchange with more compressed helium. The temperature of analysis is PID controlled (±0.001 K) from 50 to 273 K by an electric heating element uniformly distributed through the cooling surface within the analysis temperature zone.

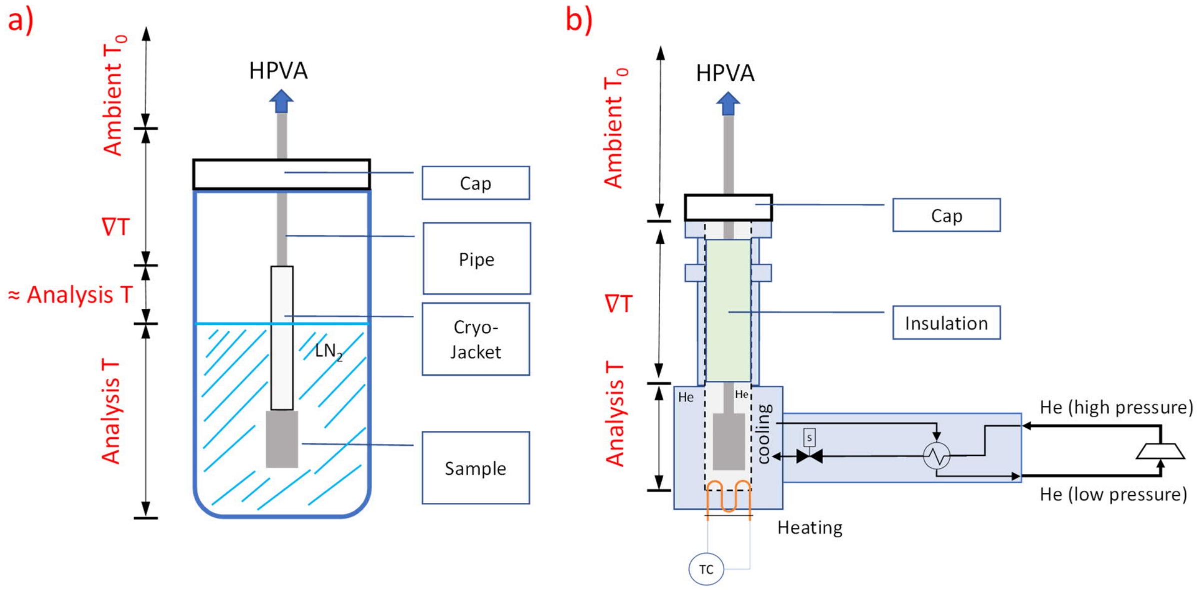

Figure S6 Vessel with liquid nitrogen (a) or cooling head (b) used to control the analysis temperature in the HPVA II.

2.3. Measurement of the free volume

The free volume (V_F) is defined as the volume of the empty sample holder minus the skeletal volume of the sample (V_{sk}) and can be directly measured by several helium expansion test at room temperature. In a volumetric experiment, the helium expansion test consists in the measure of the pressure change when a known volume of helium given by manifold volume is let to expand into the sample cell.

In the PCT-Pro the free volume is measured 5 times using a He expansion test after the sample activation. He at 7 bar is the manifold volume is allowed to expand to the sample cell and the equilibrium pressure is recorded after 20 min.

In the HPVA the free volume was measured 20 times after activation of the sample, outgassing the sample during 20 min between experiments. The accuracy of the free-space determination was tested with a non-porous SiO_2 certified material by BAM (CRM BAM – PM – 101), whose BET

area was calculated as 0.177 m²/g after N_2 adsorption-desorption at 77 K, and whose density was measured as 2.65 ± 0.01 g/cm³ by pycnometry. Activation of this material was done at 300 °C for 4 h in a dynamic vacuum (P < 10^{-6} mbar). The measurement of the free space has an error lower than 1 %.

2.4. Correction of temperature gradients

Calibration of PCT up to 25 bar

In the PCT, the temperature gradient between sample and manifold is corrected by hydrogen expansion, using a non-adsorbing material (sea sand), measured at different temperatures and pressure for various volumes of material. This yields the amount of non-adsorbed gas considering all temperatures and temperature gradients. The measurements of an adsorbing material are corrected with these values of a non-adsorbing material of the same volume i. e. the calibration accounts for the H_2 amount present in the free volume in the sample holder, with the prerequisite of knowing the skeleton volume V_{sk} of a given sample. The free volume was measured as 1.27 ± 0.02 ml. The volume of the non-adsorbing material is obtained by He test varying the amount of sea sand. For each sample investigated, V_F is measured ten times by He test. V_{sk} is obtained by subtracting V_F of analysis of the sample from the V_F of the blank experiment, this is, the empty sample cell.

$$V_{sk} = V_F^{blank} - V_F^{sample}$$

When the helium expansion test is performed at cryogenic temperatures, the measured value of the free volume at the analysis temperature is inaccurate, especially for microporous materials, due to significant helium adsorption. Besides, the calculation of the V_F at the analysis temperature considers well-defined volumes at specific temperature values (i.e., temperature of the manifold, ambient temperature, and analysis temperature). However, as shown in Figure S6a, the temperature is unknown between these sections at different temperatures, and temperature gradients are present. The actual gas densities and compressibility factors of contained gases in these regions affected by temperature gradients during the He expansion or the H_2 analysis are not considered in the adsorption uptake calculations. For these reasons, the measured adsorption isotherms need to be corrected with the corresponding calibration.

To subtract the contribution of the non-adsorbed hydrogen in the sample holder volume (V_{holder}), we measure the hydrogen uptake for a non-adsorbing reference material (sea sand) for different Vsk and temperatures. Using the reference material uptake we can calculate the so-called *'excess amount of hydrogen'*, $n_{excess}(p,T)$.

$$n_{excess}(p,T) = n_{experiment}(p,T) - n_{sea\ sand}(p,T)$$

where $n_{experiment}(p,T)$ is the raw data of hydrogen uptake measurement for the porous sample (total amount of hydrogen present in the sample container) and $n_{sea\ sand}(p,T)$ corresponds to the uptake of the sample holder with the corresponding amount of sea sand, for a given temperature and V_{sk}.

Calibration of HPVA up to 100 bar

For volumetric determination of adsorption isotherms of hydrogen at 77 K and high pressure, the amounts (n_{exc}) were calculated by subtracting the amount of gas occupying the free volume after the adsorption equilibrium (n_{eq}) to the initially dossed amount of gas (n_0). To this, it is necessary to know the volume of the manifold, the free volume at ambient temperature ($V_{F,amb}$) and the free volume at the analysis temperature ($V_{F,a}$). The V_F measured by the He test is $V_F = V_{F,amb} + V_{F,a}$.

$$n_{exc} = n_0 - n_{eq}$$

$$n_0 = \frac{P_0 \times V_m}{Z_{p_0,T_m} \times R \times T_m}$$

$$n_{eq} = \frac{P_{eq}}{R}\left(\frac{V_m}{Z_{p_{eq},T_m} \times T_m} + \frac{V_{F,amb}}{Z_{p_{eq},T_{amb}} \times T_{amb}} + \frac{V_{F,a}}{Z_{p_{eq},T_a} \times T_a}\right)$$

To consider the deviations due to the lack of linearity of the compressibility factor from low to high pressure **[4]** and those due to the evaporation of liquid nitrogen, the value of $V_{F,a}$ needs to be corrected ($C.V_{F,a}^{s}$). To calculate this corrected value, blank hydrogen adsorption experiments on the empty sample container are performed in similar conditions (analysis temperature and duration with the same empty sample cell) and up to the same pressure that those used for adsorbent samples, for which the free volume is measured (V_F^b). The value of $C.V_{F,a}^{b}$ is the value at which the adsorption uptake of the blank experiment is balanced around zero-adsorption and is calculated by an iterative

process. The corrected free volume at the analysis temperature for the experiment with adsorbent material ($C.V^{s}_{F,a}$) is calculated by the next expression:

$$C.V^{s}_{F,a} = C.V^{b}_{F,a} - \left(V^{b}_{F} - V^{s}_{F}\right)$$

Note that the difference $\left(V^{b}_{F} - V^{s}_{F}\right)$ corresponds to the volume of the sample (V_{sk}). The calculated $C.V^{b}_{F,a}$ depends on the analysis length and the ambient temperature. For this reason, blank experiments were repeated several times at ± 20 % of analysis lengths from the average value and different ambient temperatures, and the average value was used to correct the isotherms, using the standard deviation to calculate the correction error.

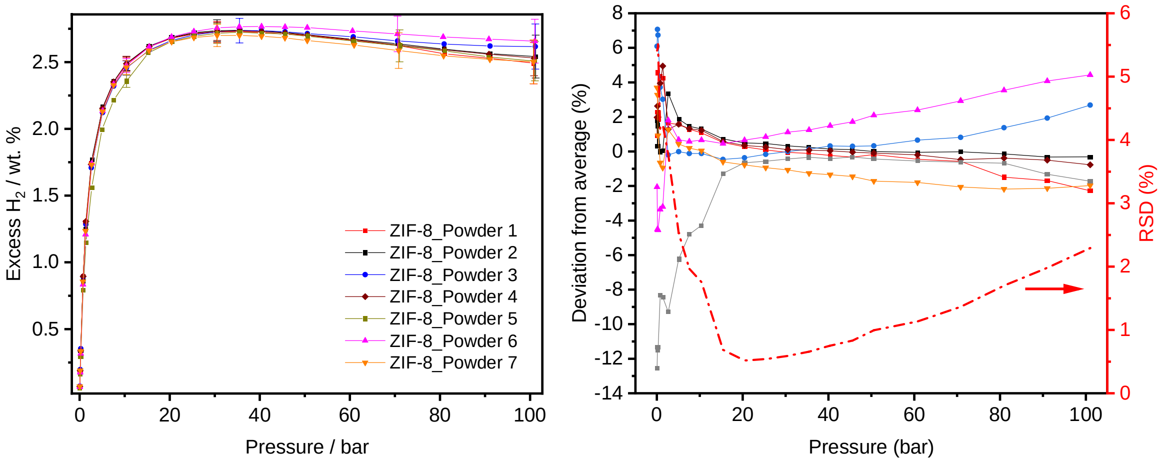

Figure S7. Comparison of excess hydrogen uptakes at 77 K of different fractions of ZIF-8 powder.

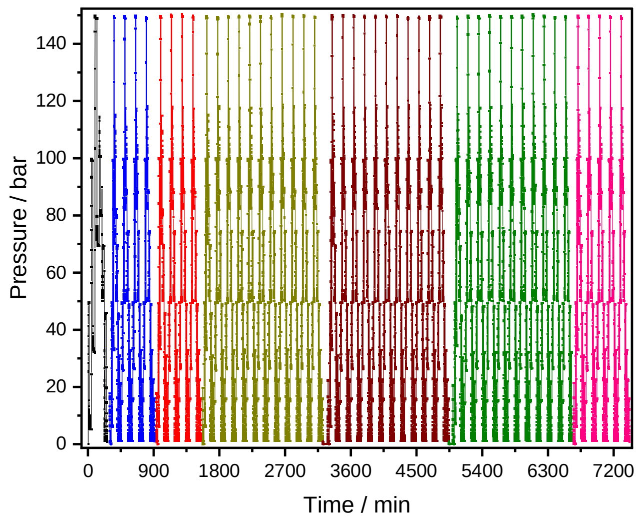

Figure S8. Pressure evolution during the cycling experiments of pelletized ZIF-8 at 77 K. Different consecutive experiments plotted in different colors.

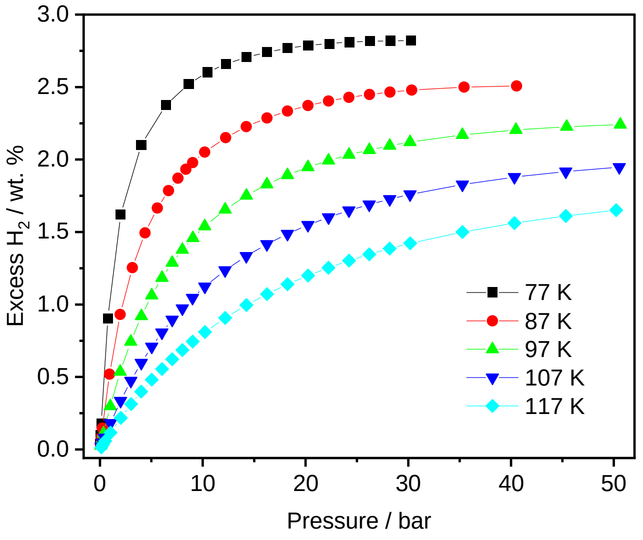

Figure S9. Excess adsorption isotherms at different temperatures of the pellets of ZIF-8.

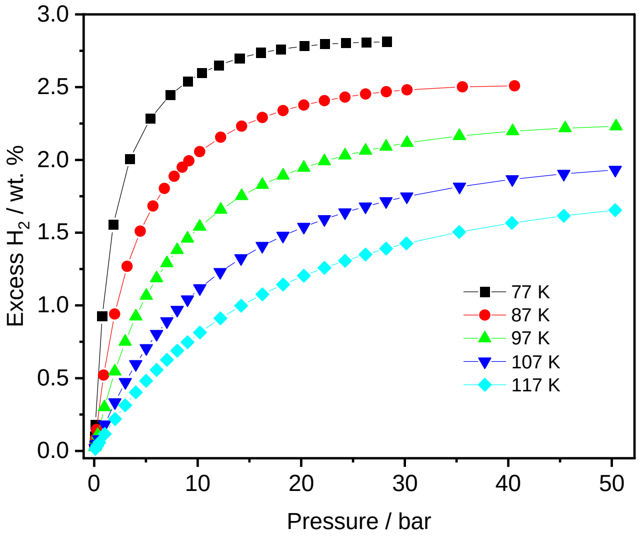

Figure S10. Excess adsorption isotherms at different temperatures of the pellets of ZIF-8 after cycling.

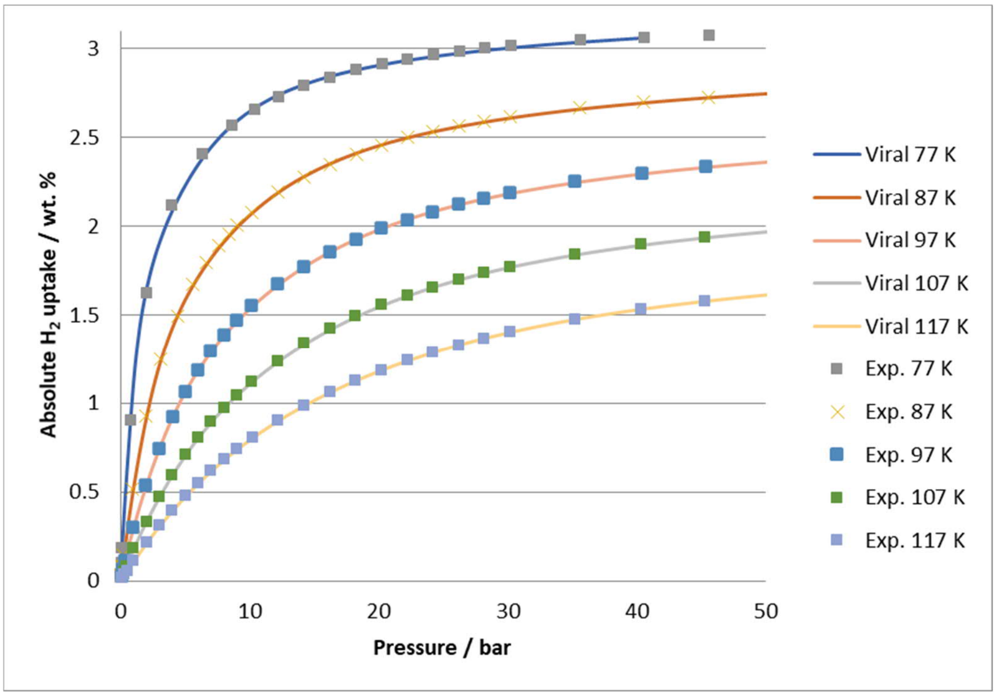

Figure S11. Absolute adsorption isotherms at different temperatures of the pellets of ZIF-8.

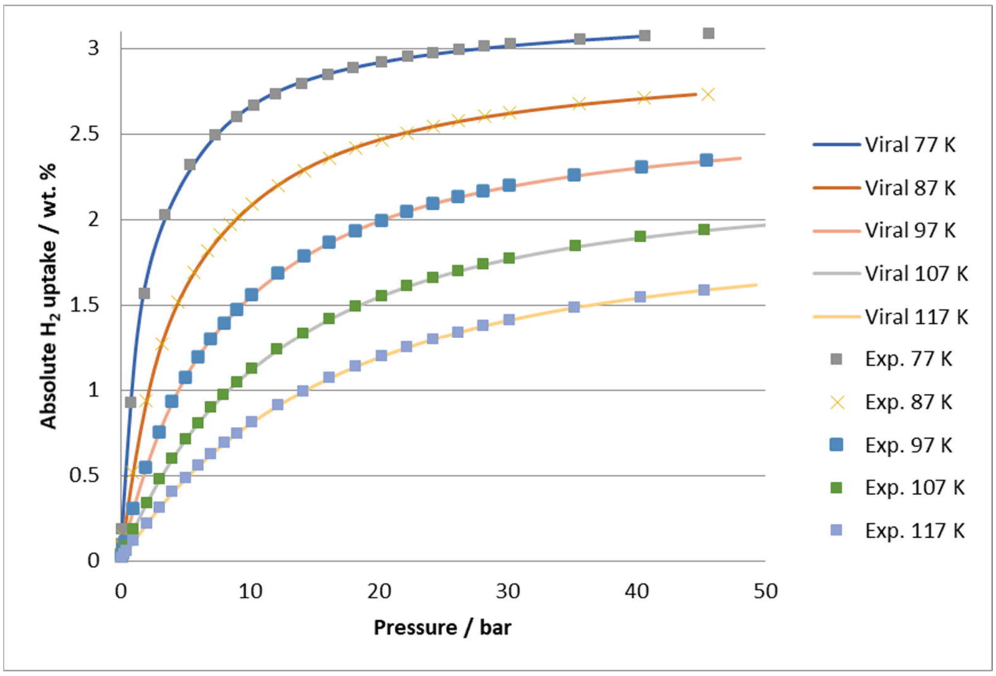

Figure S12. Absolute adsorption isotherms at different temperatures of the pellets of ZIF-8 after cycling.

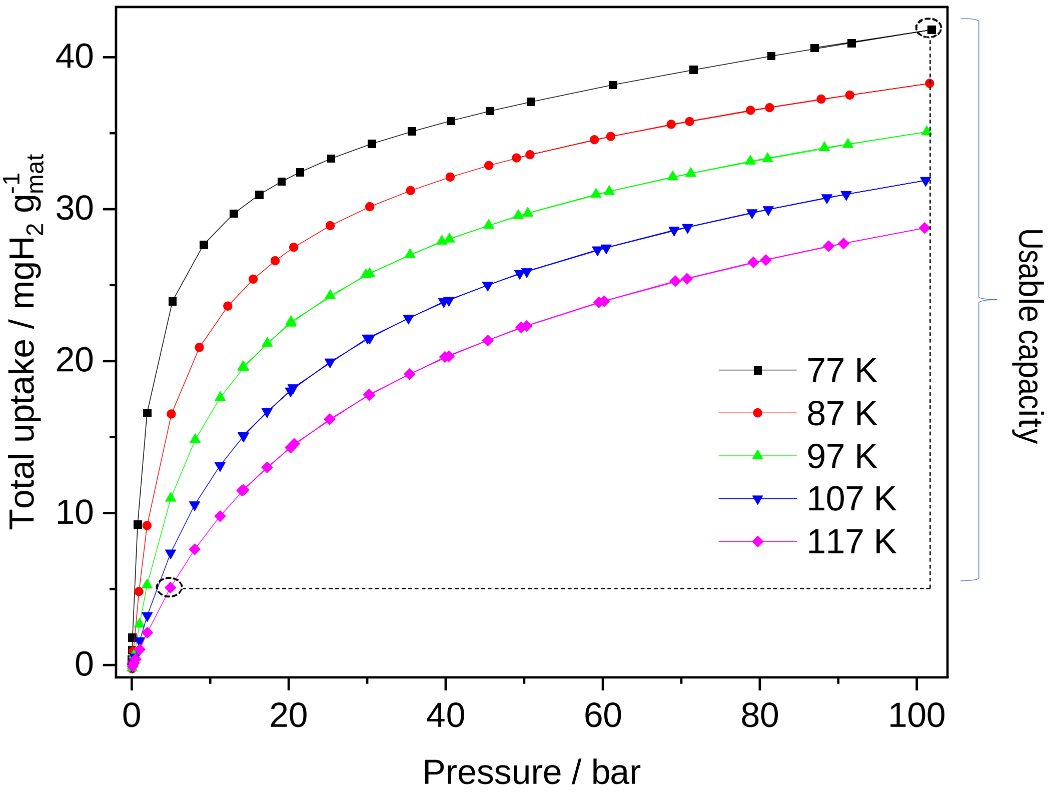

Figure S13. Calculation of the usable capacity in gravimetric units for a TSA cycle from 77 K – 100 bar and 117 K – 5 bar.

3. Measurement of hydrogen uptake by gravimetric device

3.1. Hydrogen adsorption measurements XEMIS

Figure S14 shows a schematic representation of the XEMIS device. A micro- balance inside of a chamber is connected to two detachable reactors. Two thin tungsten wires hang from both arms of the microbalance and holding the sample and counterweight. A gas inlet tube connects the pressure system to the microbalance chamber and both arms. These two arms are submerged in the bath of the refrigerant fluid so that the effect of the temperature gradients in the buoyancy of the sample is compensated with the buoyancy of the counterweight, therefore this correction is not necessary.

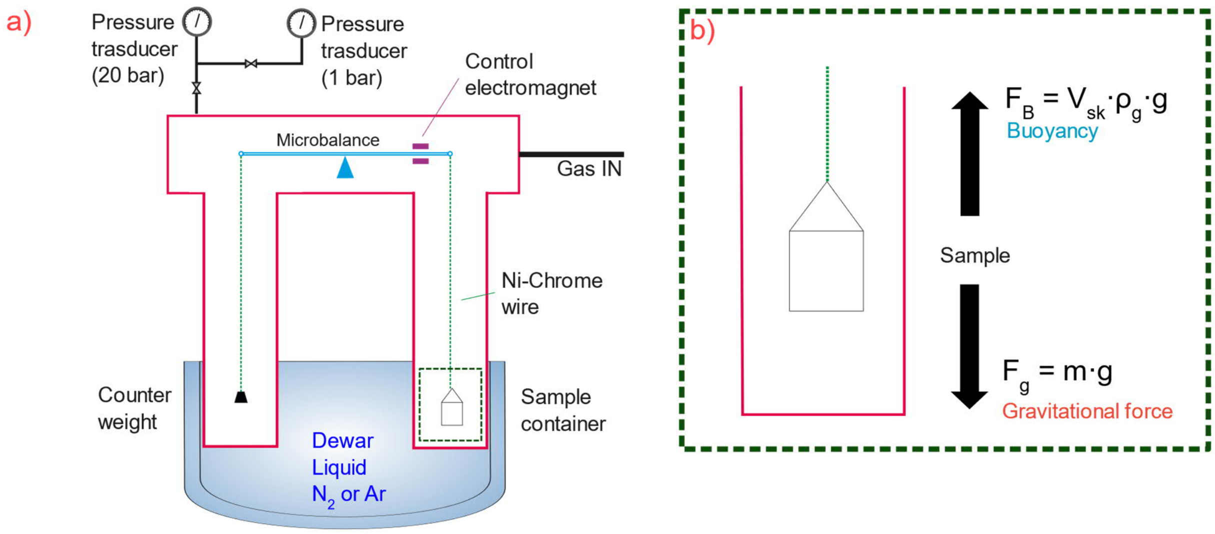

Figure S14. Microbalance diagram of the XEMIS machine. The sample holder and a counterweight are connected to the balance by a thin wire. The microbalance uses an electromagnet connected to a control unit to equilibrate the balance; the mass of the sample is proportional to the current applied to the electromagnet (a). Force diagram, buoyancy and gravity forces acting on the sample (b).

3.2. Correction of the buoyancy

For gravimetric measurements, the gravitational force F_g or weight is proportional to the sample mass and pulls down the sample with a magnitude $F_g = mg$ where g is the gravitational acceleration and m is the mass. A buoyancy force F_b pulls the sample up and it is proportional to the skeleton volume of the sample V_{sk} and to the density of the surrounding fluid ρ, and it is equivalent to the weight of the fluid that would otherwise occupy the volume of the sample $F_b = -V_{sk}\rho g$ (Figure S15). The adsorption of a fluid on the sample surface modifies the overall mass of the sample which increases the weight $\delta m_{ads} \cdot g$. A higher gas pressure increases the density of the fluid where the sample is immersed, as the density increases the buoyancy force is also higher.

The sum of all forces that the balance records as the total weight: the sample weight $m_{sample} \cdot g$, the buoyancy $V_{ske} \cdot \rho \cdot g$, and the weight change related to the gas adsorption $\delta \cdot m_{ads} \cdot g$.

$$\sum F = m_{sample} \cdot g + \delta m_{ads} \cdot g - V_{ske} \cdot \rho \cdot g$$

The correction of the buoyancy force is made using an individual He test to measure the skeleton volume of each sample. The helium test consists in measuring the buoyancy of the sample under

He atmosphere at room temperature using several pressure points. The weight of the sample against the gas density follows a negative linear tendency, for which the ratio is the skeleton volume of the sample. This test assumes that He does not interact with the surface of the sample at this temperature, meaning that any amount of He adsorbed on the sample surface will lead to some error in the subsequent assessment of adsorbed gas. The He test records the negative change in weight ΣF as function of the He pressure (Sample buoyancy). Using the state equation for He [30] and measured temperature, the He density is calculated. Figure S15 shows as an example a typical He test result for ZIF-8 (Pellets).

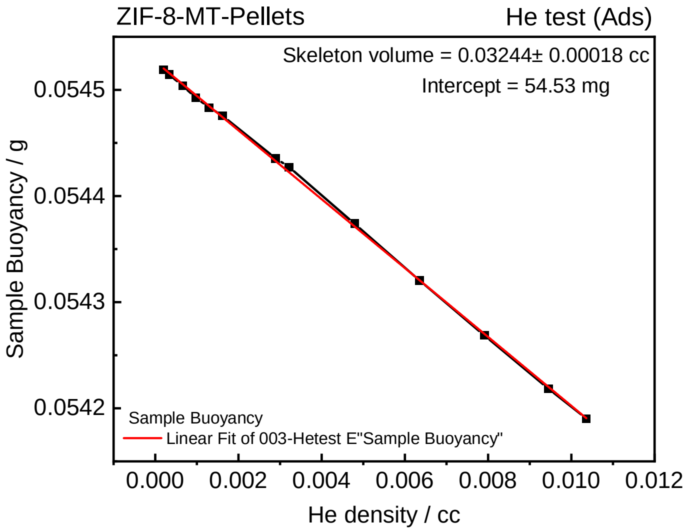

Figure S15. Skeleton volume measured by He test. The skeleton volume of the sample is the slope of the linear fitting to the buoyancy vs the gas density.

3.3. Correction of the temperature gradient in XEMIS

Analogous to volumetric devices, the thermal gradients between sections of the gravimetric apparatus also affect the measurement of adsorption results. The thermal gradient generates a volume where the gas density is unknown, therefore the correction of the buoyancy is inaccurate and needs a calibration performing He tests of non-adsorbing samples with the same volume than the adsorbing sample. However, the XEMIS apparatus used herein is not affected by the thermal gradient because the temperature of both the sample and the counterweight arms is controlled with the same cooling system. Therefore, the deviation of the buoyancy correction is the same in both sides, compensating one for the other.

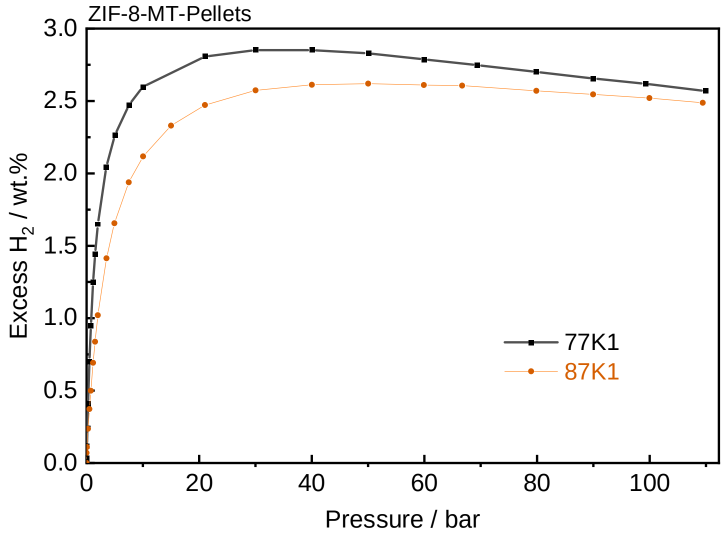

Figure S16. Excess and absolute hydrogen adsorption isotherms on pelletized material ZIF-8 measured with the XEMIS device.

4. Calculation of the heat of adsorption:

The heat of adsorption (Q_{st}) can be calculated from isothermal adsorption-desorption data collected at different temperatures by using the *Clausius-Clapeyron* equation.

$$Q_{st} = -\mathrm{R} \cdot \left(\frac{\partial \ln P}{\partial {}^{1}/_{T}} \right)_{\mathrm{n_a}}$$

In this work, hydrogen adsorption experiments were performed at up to five different temperatures (77, 87, 97, 107, 117 K). A van´t Hoff diagram (Figure S17) can be plotted from equilibrium pressure and temperature for the same amount of adsorbed gas (isosteres) and linearly least-squares fitted to calculate the heat of adsorption from each line's slopes. In order to provide n_a-T-P_{eq} data between experimental points, interpolation within each adsorption isotherm can be performed linearly or by fitting the isotherms to the EOS of Virial.

$$\mathrm{Ln(P)} = \mathrm{Ln(N)} + \frac{1}{T}\sum_{i=0}^{m} a_i \cdot N^i + \sum_{i=0}^{n} b_i \cdot N^i$$

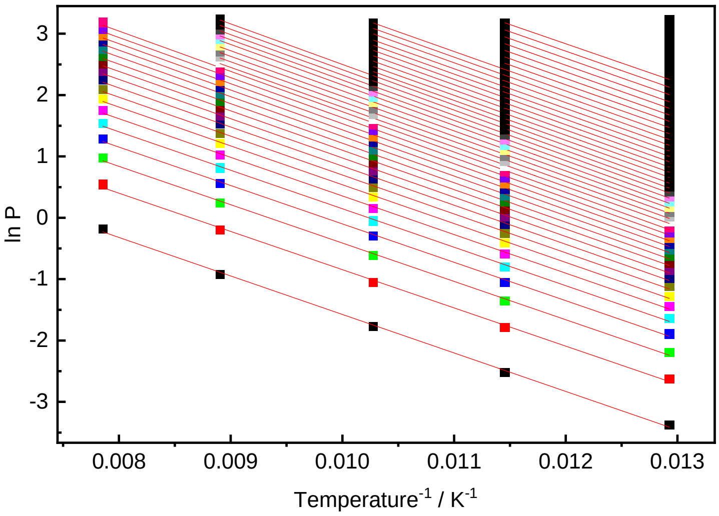

Figure S17. Van´t Hoff diagram used to calculate the isosteric heat of adsorption of the powder ZIF-8.

References

1. Blach, T.P. and E.M. Gray, *Sieverts apparatus and methodology for accurate determination of hydrogen uptake by light-atom hosts.* Journal of Alloys and Compounds, 2007. 446: p. 692-697.
2. Leachman, J.W., et al., *Fundamental Equations of State for Parahydrogen, Normal Hydrogen, and Orthohydrogen.* Journal of Physical and Chemical Reference Data, 2009. 38(3): p. 721-748.
3. NIST. *Thermophysical Properties of Fluid Systems. Chemistry WebBook, SRD 69*. 2022 [cited 2022 3/10/2022]; Available from: https://webbook.nist.gov/chemistry/fluid/.
4. Zhou, L. and Y.P. Zhou, *Determination of compressibility factor and fugacity coefficient of hydrogen in studies of adsorptive storage.* International Journal of Hydrogen Energy, 2001. 26(6): p. 597-601.
